# Supplementary material for: Wettability of Amino Acid-Functionalized PSMA Electrospun Fibers for the Modulated Release of Active Agents and Its Effect on Their Bioactivity
Source: Pharmaceutics. 2023 Jun 5;15(6):1659. doi: 10.3390/pharmaceutics15061659 (PMC10301643; doi:10.3390/pharmaceutics15061659)
Supplement: Supplementary file 1 [file pharmaceutics-15-01659-s001.zip › pharmaceutics-2399179-supplementary.pdf]

pharmaceutics

Type of the Paper (Article)

Wettability of Amino Acid-functionalized PSMA Electrospun fibers for the Modulated Release of Active Agents and its Effect on their Bioactivity

Sebastián Santander [1], Nicolás Padilla-Manzano [1], Bastián Díaz [1], Renato Bacchiega [1], Elizabeth Jara [1], Luis Felipe Álvarez [1], Cristóbal Pinto [1], Juan C. Forero [2], Paula Santana [3], Eugenio Hamm [4], Marcela Urzúa [1]*, Laura Tamayo [1]*

[1] Departamento de Química, Facultad de Ciencias, Universidad de Chile, Las Palmeras 3425, Santiago, Chile.
[2] Escuela de Ciencias de la Salud, Universidad de Viña del Mar. Cod. postal: 2572007. Viña del Mar, Valparaíso, Chile.
[3] Instituto de Ciencias Químicas Aplicadas, Facultad de Ingeniería, Universidad Autónoma de Chile, El Llano Subercaseaux 2801, San Miguel, Santiago 8910060, Chile.
[4] Departamento de Física, Facultad de Ciencia, Universidad de Santiago de Chile, av. Víctor Jara 3493, Estación Central, Santiago, Chile
* Correspondence: laura.tamayo@uchile.cl, maurzua@uchile.cl.

Keywords: Fibers; functionalization; amino acid; electrospinning; wettability; release.

Citation: To be added by editorial staff during production.

Academic Editor: Firstname Lastname

Received: date
Revised: date
Accepted: date
Published: date

Copyright: © 2023 by the authors. Submitted for possible open access publication under the terms and conditions of the Creative Commons Attribution (CC BY) license (https://creativecommons.org/licenses/by/4.0/).

1. Supplementary Material

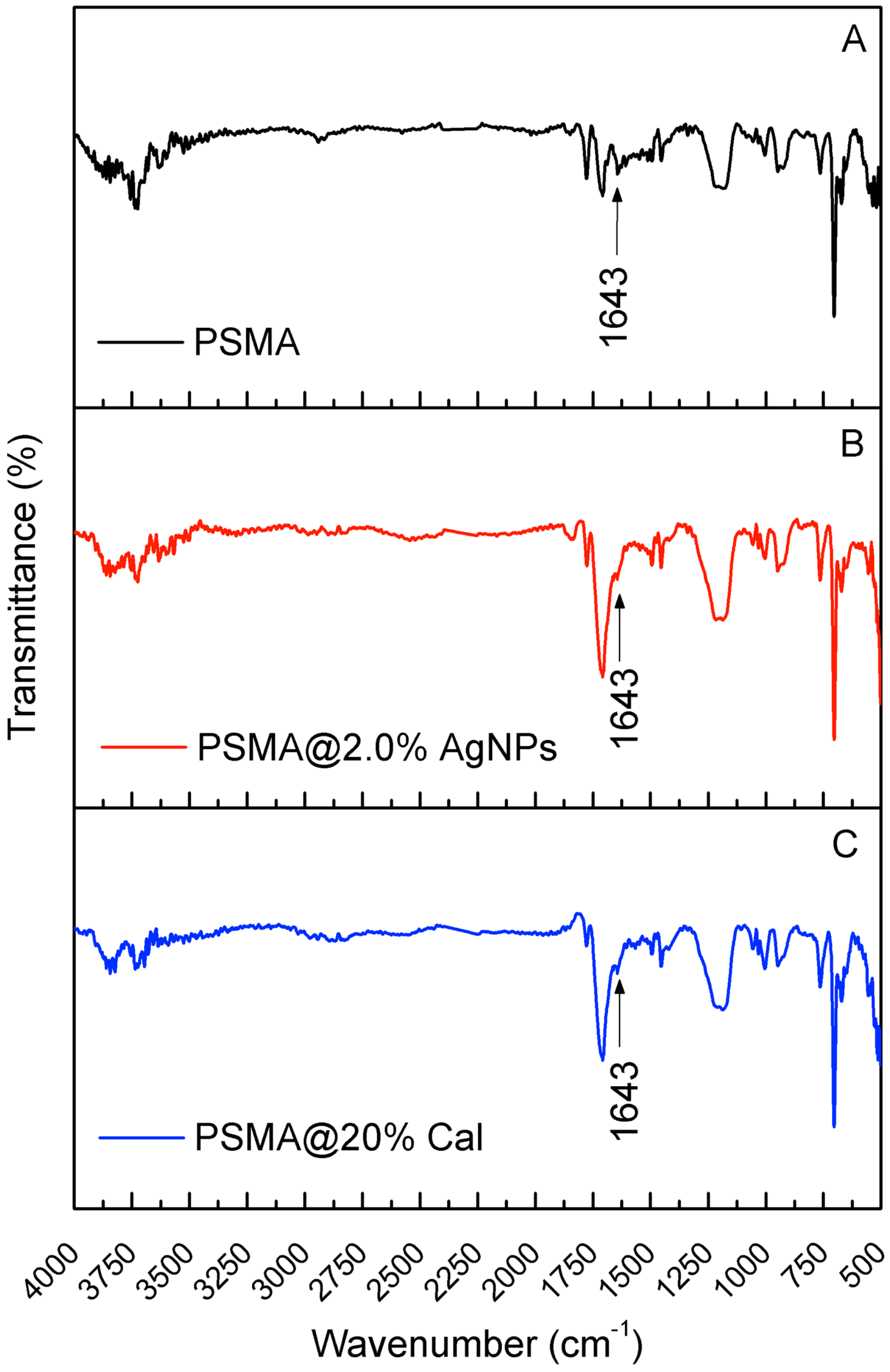

Figure S1. ATR-FTIR spectra of mats of (a) PSMA, (b) PSMA-AgNPs (2.0 wt.%) and (c) PSMA-Cal (20 wt.%).

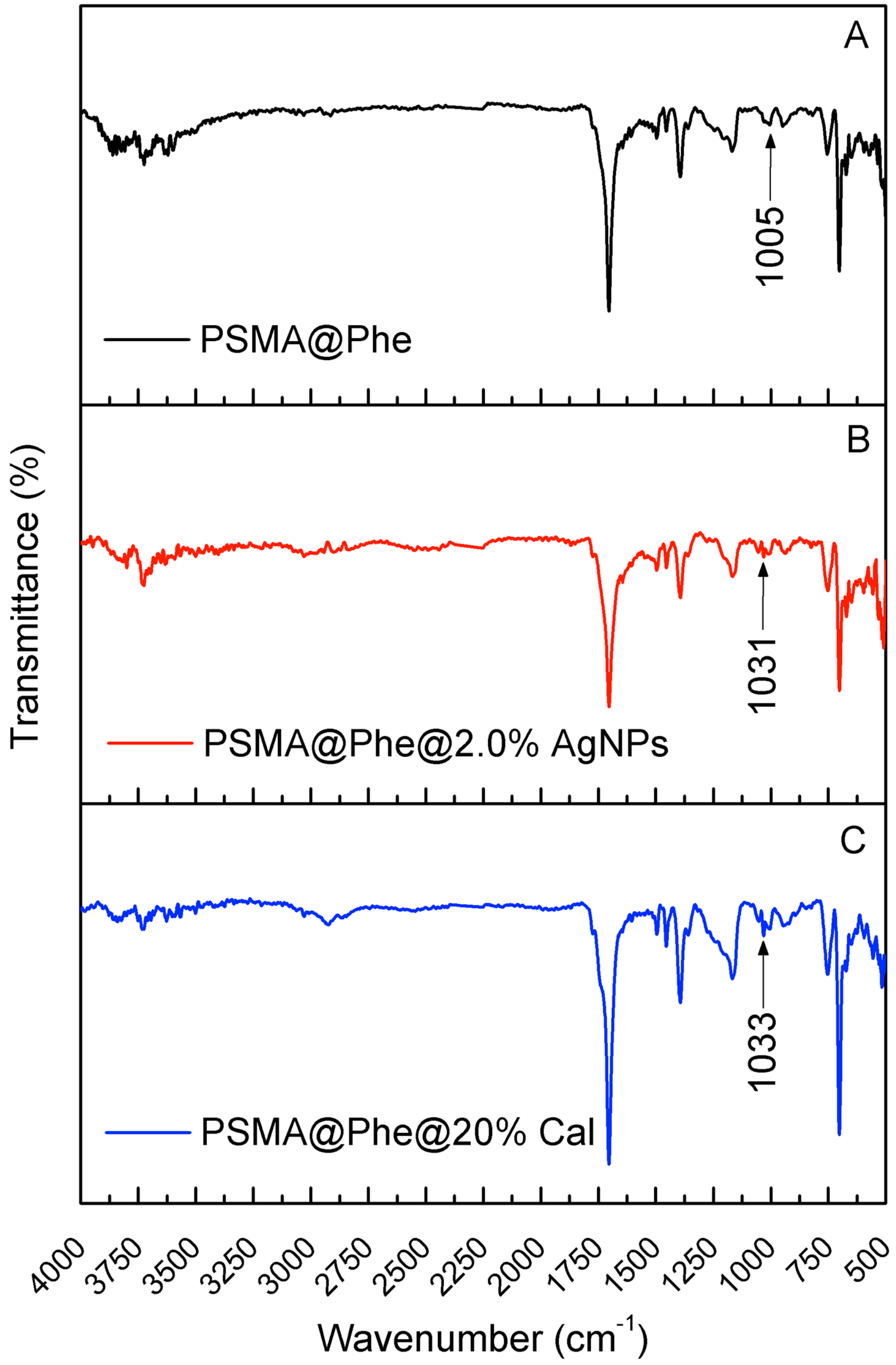

Figure S2. ATR-FTIR spectra of mats of (a) PSMA@Phe, (b) PSMA@Phe-AgNPs (2.0 wt.%) and (c) PSMA@Phe-Cal (20 wt.%).

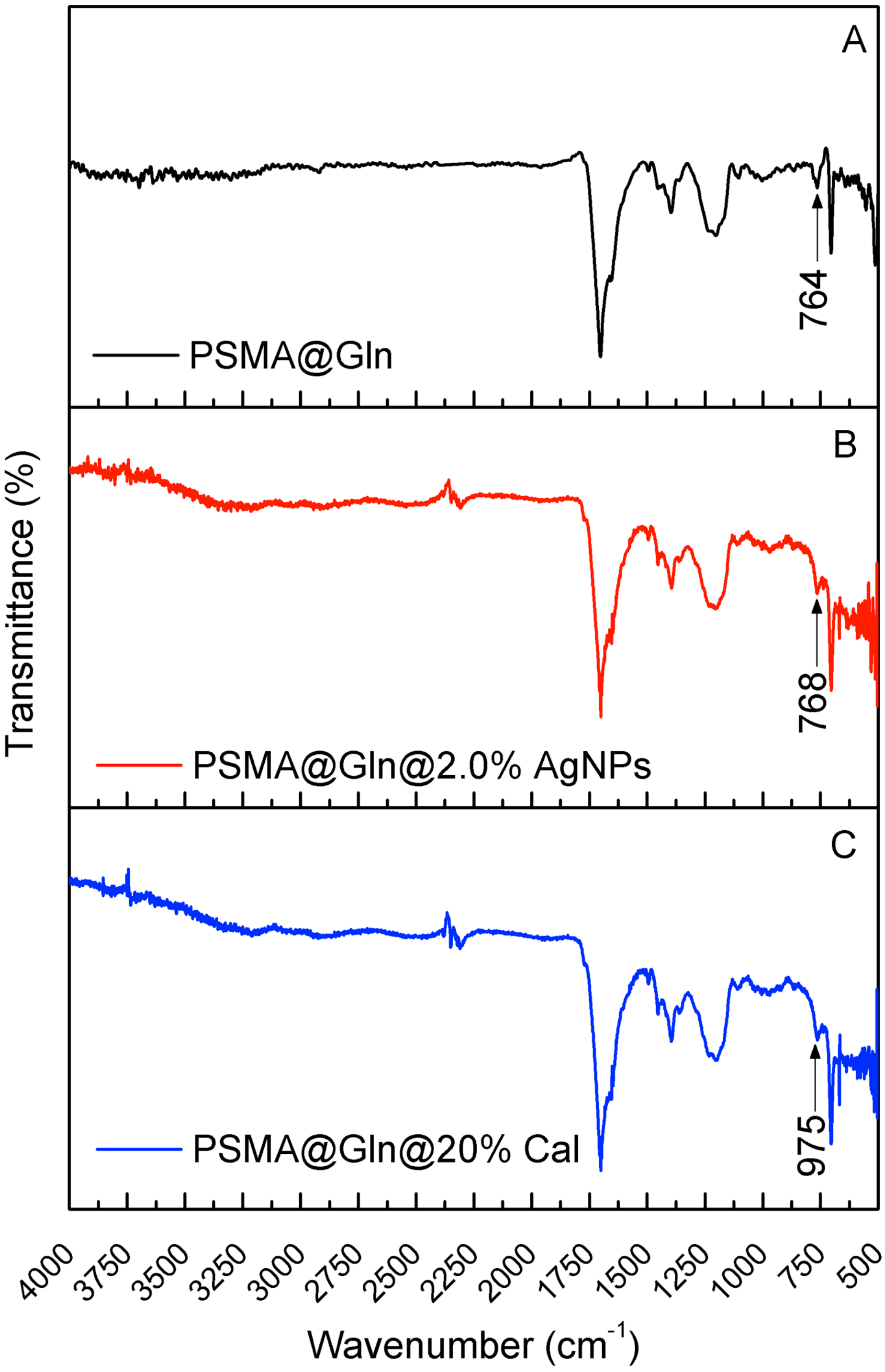

Figure S3. ATR-FTIR spectra of mats of (a) PSMA@Gln, (b) PSMA@Gln-AgNPs (2.0 wt.%) and (c) PSMA@Gln-Cal (20 wt.%).

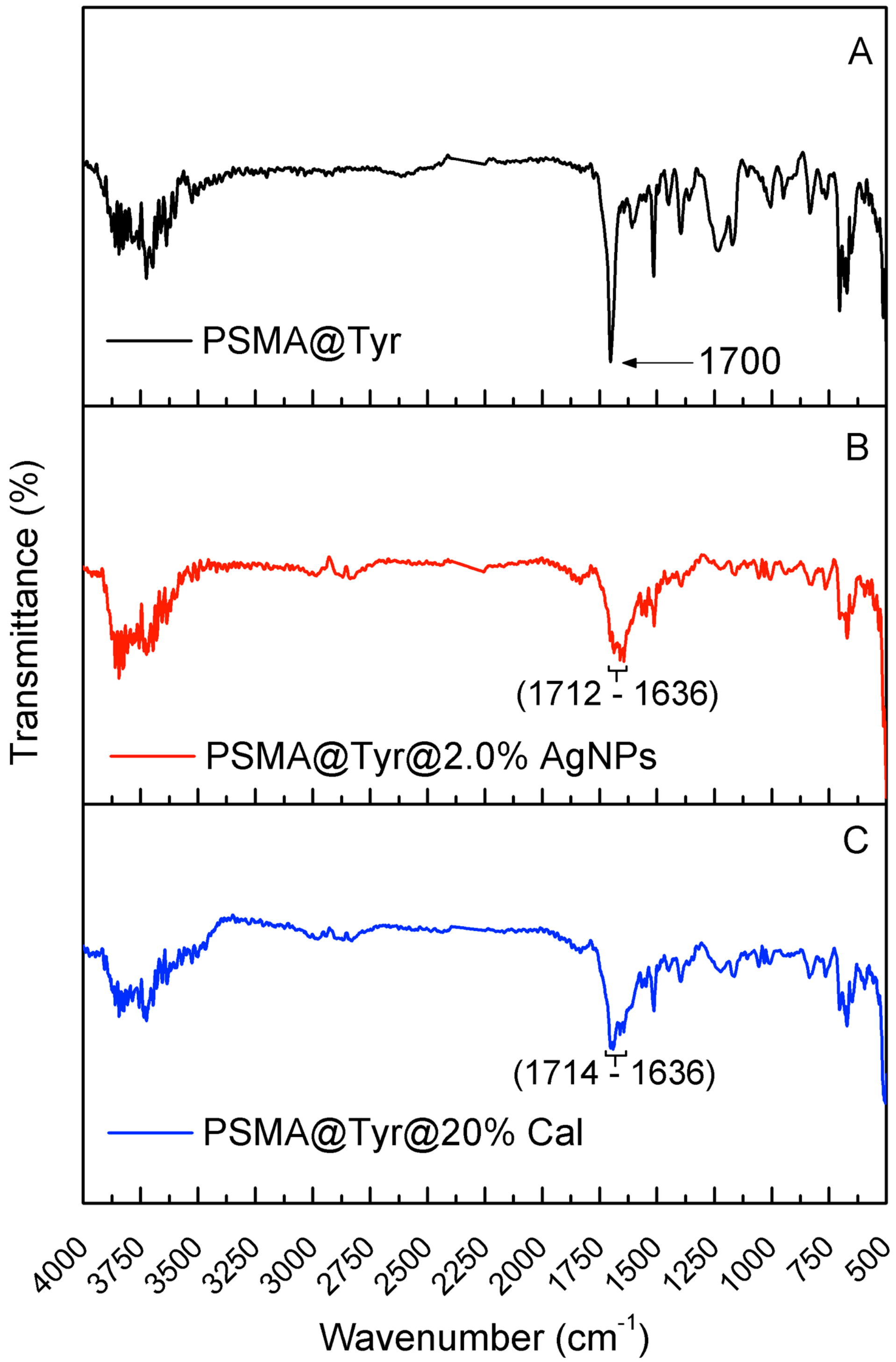

Figure S4. ATR-FTIR spectra of mats of (a) PSMA@Tyr, (b) PSMA@Tyr-AgNPs (2.0 wt.%) and (c) PSMA@Tyr-Cal (20 wt.%).

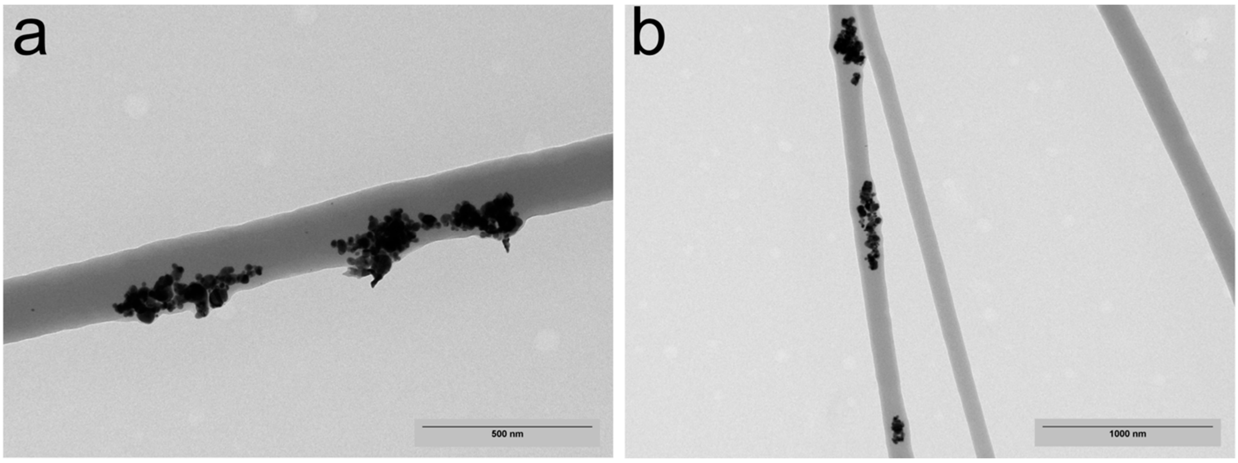

Figure S5. PSMA and PSMA@Phe fibers containing AgNPs aggregates. (a) PSMA 2.0 wt.%, (b) PSMA@Phe 2.0 wt.%.

Table S1. Advancing, receding and hysteresis contact angle of PSMA mats with different concentrations of active agent.

	**Active Agent (wt.%)**	**Advancing contact angle (°)**	**Receding contact angle (°)**	**Hysteresis (°)**
PSMA	-	132.7	116.4	16.3
	5 cal	124.6	90.3	34.3
	10 cal	133.4	124.5	8.9
	15 cal	133.5	127.2	6.3
	20 cal	134.3	125.0	9.3
	0.5 AgNPs	127.2	114.0	13.2
	1.0 AgNPs	137.6	113.1	24.5
	1.5 AgNPs	130.6	108.6	22.0
	2.0 AgNPs	123.0	106.5	16.5

Table S2. Advancing, receding and hysteresis contact angle of PSMA@Phe mats with different concentrations of active agent.

	**Active Agent (wt%)**	**Advancing contact angle (°)**	**Receding contact angle (°)**	**Hysteresis (°)**
PSMA@Phe	-	136.8	129.5	7.3
	5 cal	135.7	127.8	7.9
	10 cal	130.2	122.4	7.8
	15 cal	134.5	126.3	8.2
	20 cal	134.0	125.1	8.9
	0.5 AgNPs	130.0	120.2	9.8
	1.0 AgNPs	129.8	122.7	7.1
	1.5 AgNPs	132.1	124.9	7.2
	2.0 AgNPs	126.1	122.5	3.6

Table S3. Advancing, receding and hysteresis contact angle of PSMA@Tyr mats with different concentrations of active agent.

	**Active Agent (wt%)**	**Advancing contact angle (°)**	**Receding contact angle (°)**	**Hysteresis (°)**
PSMA@Tyr	-	130.4	117.9	12.5
	5 cal	127.4	114.3	13.1
	10 cal	137.4	118.0	19.4
	15 cal	130.6	119.6	11.0
	20 cal	134.1	123.1	11.0
	0.5 AgNPs	128.3	115.0	13.3
	1.0 AgNPs	77.2	34.0	43.2
	1.5 AgNPs	40.6	30.1	-
	2.0 AgNPs	135.5	65.5	70.0

Table S4. Contact angle at 2 different times, t=0 s and t=6 s of the PSMA@Gln mats with different concentrations of active agent.

	**Active Agent (wt%)**	**t_0 contact angle (°)**	**t_1 contact angle (°)**
PSMA@Gln	-	28.7	25.5
	5 cal	29.7	19.8
	10 cal	41.5	28.1
	15 cal	82.6	46.0
	20 cal	32.1	26.0
	0.5 AgNPs	22.9	20.8
	1.0 AgNPs	36.2	35.6
	1.5 AgNPs	27.7	24.2
	2.0 AgNPs	32.7	28.9

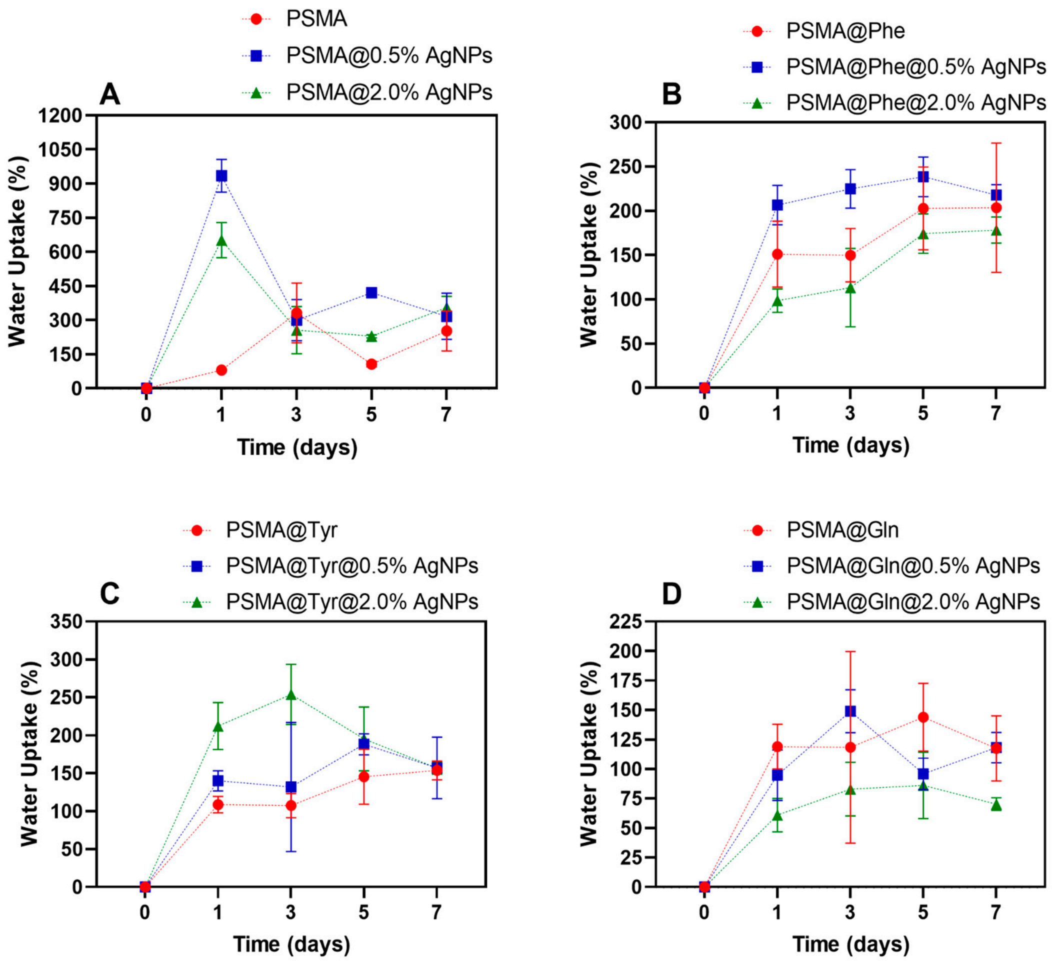

Figura S6. Water absorption capacity of PSMA and PSMAf containing concentrations of AgNPs at 0.5 and 2.0 wt.% (a) PSMA, (b) PSMA@Gln, (c) PSMA@Tyr and (d) PSMA@Phe.

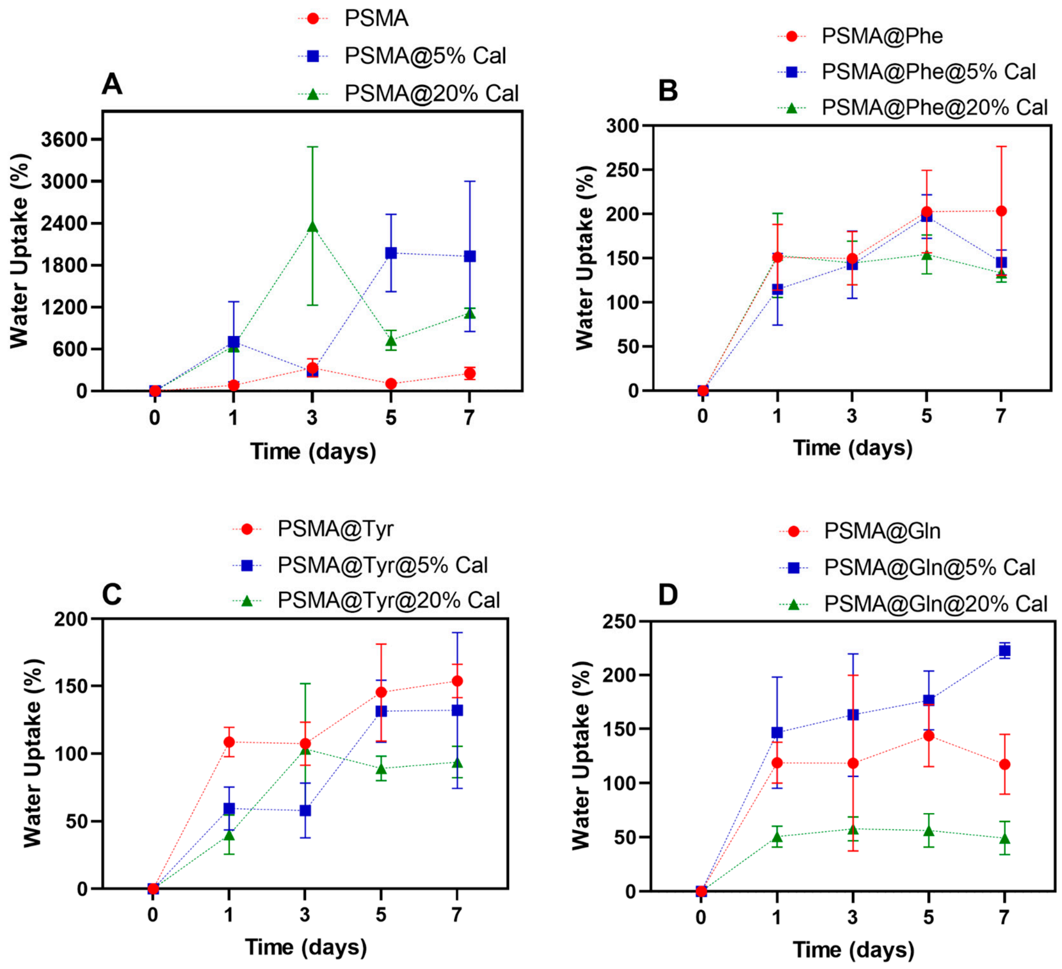

Figura S7. Water absorption capacity of PSMA and PSMAf containing *C. officinalis* concentrations at 5 and 20 wt.% (a) PSMA, (b) PSMA@Gln, (c) PSMA@Tyr and (d) PSMA@Phe.

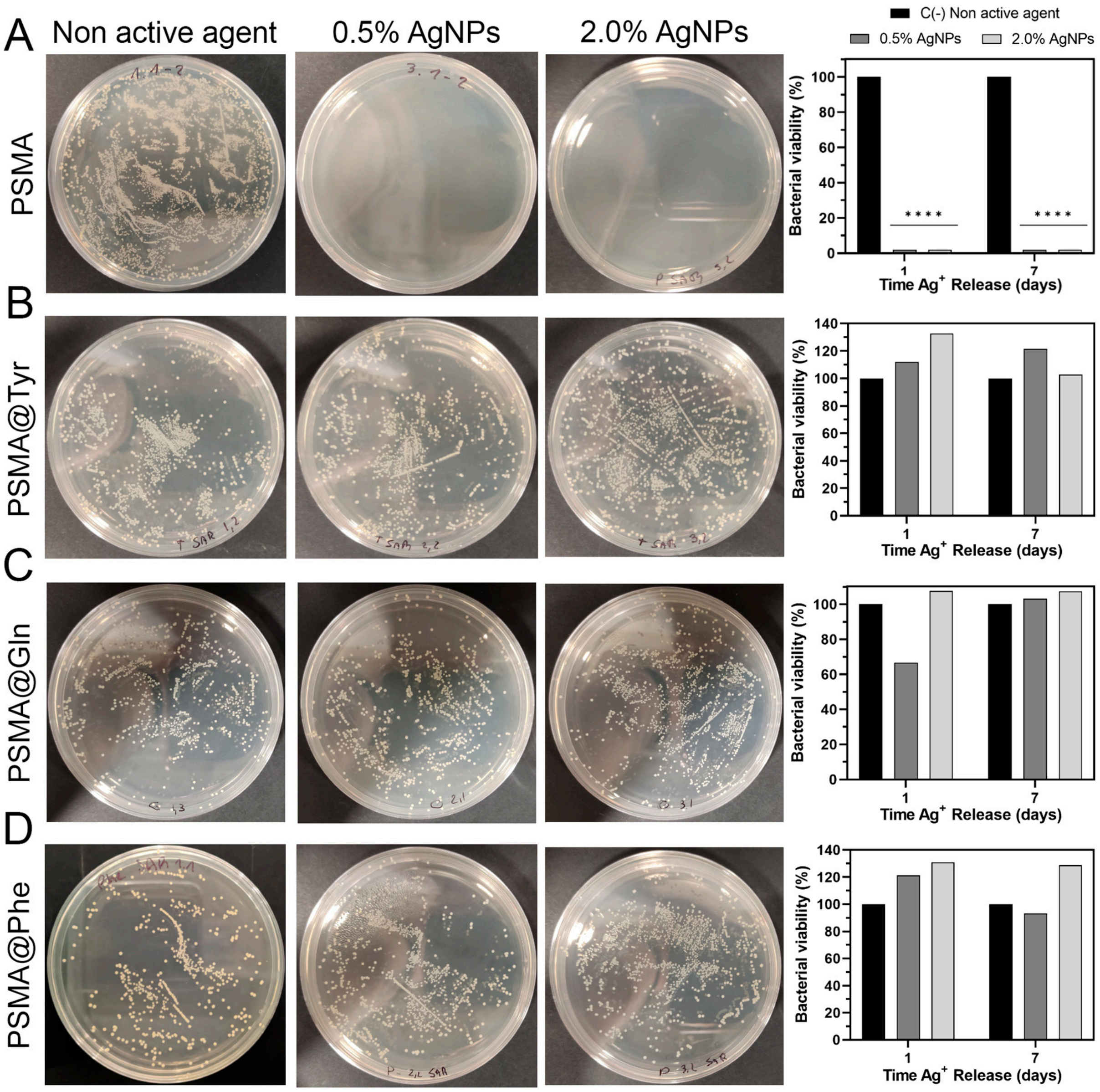

Figura S8. Bacterial viability of methicillin-resistant *S. aureus* ATCC 33592 incubated in release solutions of, (a) PSMA, (b) PSMA@Gln, (c) PSMA@Tyr and (d) PSMA@Phe. Data were analyzed using Dunnett's multiple comparisons test; **** $P < 0.0001$ compared to the negative control group (Non-active agent).
